# A Movement Description Language for Functional Training Exercise Analysis

**DOI:** 10.3390/jfmk11020162

**Published:** 2026-04-21

**Authors:** Lúcia Sousa, Daniel Canedo, Pedro Santos, António Neves

**Affiliations:** 1 Institute of Electronics and Informatics Engineering of Aveiro, Department of Electronics, Telecommunications and Informatics, University of Aveiro, 3810-193 Aveiro, Portugal; luciamsousa00@ua.pt; 2 MARE Box, 3830-552 Gafanha da Nazaré, Portugal; pedro.ricardo.ferreira.santos@gmail.com

**Keywords:** movement description language, exercise composition, exercise repetition detection, movement analysis, functional training, pose estimation

## Abstract

**Objective:** Functional training exercises involve complex multi-joint movements that challenge traditional rule-based or data-driven recognition systems. This paper introduces a Movement Description Language (MDL) designed to formally represent, analyze, and evaluate such exercises using camera-based pose estimation and interpretable, composable structures. **Methods:** The proposed MDL models each exercise as a finite-state machine defined by pose-derived angle proxy transitions, allowing movements to be described in a modular and reusable way. Demonstrated with MediaPipe landmark extraction from monocular video, while the MDL remains compatible with any pose estimation algorithm, the framework focuses on exercise phase detection and repetition counting. Experimental validation was conducted on a dataset of 1513 videos of 12 functional exercises (squats, deadlifts, lunges, shoulder presses, planks, push-ups, pull-ups, bent-over rows, box jumps, thrusters, overhead squats, and burpees) obtained from public pose datasets, competition footage, and recordings of 9 participants in real-world environments. **Results:** Automated repetition counts were compared against manually annotated ground truth, showing an overall repetition-counting accuracy of 97.2%, with a mean per-exercise accuracy of 98.8% (range 95–100%). The MDL successfully handled both simple and compound exercises, maintaining reliable phase detection despite variations in execution speed, camera perspective, and environmental conditions. **Conclusions:** The system was implemented using real-time pose estimation to demonstrate the practical execution of the MDL framework. The proposed MDL provides a transparent, extensible, and computationally efficient framework for functional exercise analysis. By bridging human-readable movement semantics with executable motion logic, it enables interpretable automatic repetition counting and phase detection, offering an alternative to black-box recognition approaches. The results support its potential for scalable deployment in training, monitoring and movement analysis applications. The proposed system is not intended for biomechanical measurement or clinical-grade kinematic analysis, but rather for interpretable modeling of exercise structure and repetition detection using approximate pose-derived signals.

## 1. Introduction

In recent years, the integration of computer vision with biomechanics has greatly advanced the capacity to analyze and optimize human movement, creating new opportunities to personalize sports training and enhance injury prevention methodologies [1]. Through computer vision and automated data interpretation, it is increasingly possible to extract approximate kinematic signals such as pose-derived angle proxies from video recordings. This enables real-time monitoring of movement patterns including joint angles, speed, and exercise form, supporting fitness applications such as repetition counting and technique feedback [2].

Functional training (FT), emphasizes compound, multi-joint exercises and integrated movement patterns crucial to health, athletic performance, and rehabilitation, continues to represent a major challenge for rule-based and data-driven recognition systems [1,3].

FT enhances athletic performance (sprinting, jumping) and daily function by addressing deficiencies in traditional single-joint training through fundamental patterns, like, squatting, lunging, hinging, pushing, pulling, carrying, often combined into complex exercises like thrusters or burpees [4,5].

The Functional Movement Screen (FMS) assesses these via seven tests, revealing asymmetries for targeted programming [5]. Biomechanically, various exercises are defined by joint angles: squats (∼113° knee/128° hip at parallel) [6]; deadlifts (hip 66–149°, knee 57–95°) [7]; and push-ups (elbow 90–180°) [8].

Several frameworks describe human motion, but none fully support computational FT analysis:

- Movement notation systems like Labanotation [9] and Eshkol-Wachman Movement Notation (EWMN) [10] provide detailed symbols for spatial/temporal body-part documentation. While applied in sports/rehabilitation, they require manual expert transcription and lack computational integration for real-time pose data.
- Motion grammars infer syntactic structures from motion data, learning elementary actions and hierarchical rules via context-free grammars, the Common Morphokinetic Alphabet (CMA) formalizes visual movement syntax [11]. These capture patterns but are not exercise-specific or sensor-driven.
- Motion description languages include MotionScript, generating natural language from 3D poses/temporal dynamics [12], and the 4-Element Model (motion/force/control/energy) for therapy [13].
- Skeleton-based action recognition (SBAR) uses graph convolutional networks (GCNs) on key points to identify exercises [14]. SU-EMD covers strength exercises with one-shot GCNs, achieving 87.4% accuracy [14]. Temporal modeling handles cycles via RepNet [15], joint angles via BiLSTM [16], and spatio-temporal graphs via ST-GCN/ST-VGCN [17].

As athletes and clinical populations increasingly seek adaptive, feedback-driven routines tailored to individual biomechanics, there is a rapidly growing demand for frameworks precisely describing, evaluating, and tracking movement across diverse contexts, from elite sports to physiotherapeutic rehabilitation [18]. Critical gaps persist:

1. SBAR/GCNs require large labeled datasets per exercise, lack interpretability [19];
2. Temporal models struggle with novel compositions;
3. Notations/grammars remain manual/non-executable;
4. No composable language encodes FT phases/transitions for real-time pose analysis.

This work introduces a Movement Description Language (MDL) as an executable domain-specific language for representing functional training exercises through pose-derived angle proxies. Unlike existing motion grammars and notation systems, which remain descriptive and require manual interpretation, MDL compiles human-readable exercise definitions into runtime finite-state machines that operate directly on pose estimation outputs. In contrast to rule-based exercise recognition systems, MDL explicitly formalizes composability, enabling the construction of complex exercises from reusable primitive modules without redesigning the system. Furthermore, unlike skeleton-based action recognition approaches that depend on large labeled datasets and model retraining, MDL generalizes to new exercise compositions through parameterized definitions rather than learned representations. This establishes a direct mapping between interpretable movement semantics and executable motion logic, supporting real-time phase detection and repetition counting without a training stage.

The objective of this study is to introduce a language for encoding functional training exercise phases using camera-only visual data, tested with MediaPipe landmark extraction while remaining compatible with any pose estimation algorithm. This approach enables users to model exercises—such as squats, deadlifts, and compound movements like thrusters, as modular finite-state machines driven by pose-derived angle proxies from monocular video pose estimation. The MDL provides interpretable exercise definitions that support efficient phase detection and repetition counting in practical training environments. Unlike existing systems, MDL provides an executable specification that directly maps human-readable movement definitions to real-time phase detection without requiring model training or post-hoc interpretation.

The MDL provides a computational representation of movement based on explicit pose-derived angle proxies, state transitions, and logical conditions. Each exercise is defined as a finite-state machine, where the states correspond to movement phases, and transitions are triggered by measurable pose-derived angle proxies derived from pose estimation landmarks. This approach enables exercises to be modularly constructed, allowing complex movements (such as thrusters or burpees) to be represented as compositions of simpler fundamental exercises (e.g., squats, pushes, planks). The MDL framework was tested in a real-time vision-based system that uses MediaPipe for pose tracking, demonstrating how formally defined motion logic can directly guide automated repetition counting and exercise phase recognition. This establishes a transparent bridge between human-readable exercise definitions and executable motion analysis, independent of the underlying pose estimation technology. It is important to emphasize that the proposed MDL is not designed as a biomechanical measurement framework. Instead, it provides a formal and interpretable representation of exercise structure based on approximate pose-derived signals, enabling practical movement analysis in real-world settings.

This paper is organized as follows: Section 2 describes the materials and methods; Section 3 details the results; Section 4 discusses the results and future directions; and Section 5 presents the conclusions and future work.

## 2. Materials and Methods

### 2.1. Movement Description Language

Pose-derived angle proxies capture position information during movements. These proxies are rarely applied in machine learning and data science compared to skeleton data despite their advantages; for example, the joint angles are viewpoint- and subject-independent, individual movements are decoupled, and the angles at each joint are independent of the angles elsewhere [20]. Pose-derived angle proxies are promising for applications where a human expert, such as a coach, has to interact with software and communicate poses and movements in terms of joint angles, as they enable more natural interaction and communication [20]. Combined with velocity estimates, they provide comprehensive performance measurement.

Prior research demonstrates that strength training outcomes vary systematically with joint position [21]. For squats, published studies report meaningful differences across knee flexion angles such as $70^{\circ }$, $90^{\circ }$, and $100^{\circ }$, while biomechanical reviews emphasize that squat depth is commonly evaluated using approximate joint angle ranges rather than a single fixed value [22] and that deadlift knee flexion values are around $103^{\circ }$ and $108^{\circ }\;\pm \;22^{\circ }$ [23]. In addition, monocular pose estimation introduces a non-negligible joint angle error, with the reported knee flexion error ranging from 9.3° to 25.8° depending on the model and setting [24]. Accordingly, our default angle thresholds (15–30° tolerance) are informed by established exercise science ranges. This design allows the system to accommodate both pose estimation uncertainty and inter-individual variability, including users with reduced mobility or movement limitations. The thresholds remain user-adjustable to support different execution styles and subject-specific range of motion.

The proposed MDL formally models each exercise as a finite state machine with explicitly defined states, transitions, and threshold parameters. The MDL adopts best practices from formal modeling (e.g., UML/Statecharts and Modelica state machines) and movement analyses (e.g., Labanotation and MotionScript) to describe human motion phases in a concise, composable way. Each exercise is declared as an MDL module containing the following:

- States—distinct movement phases (for example, in a squat, start, descending, and ascending).
- Transitions—how the movement changes from one phase to another, based on conditions like specific pose-derived angle proxies.
- Parameters or thresholds—numerical limits such as pose-derived angle proxies or durations that trigger these transitions.

The functional movement patterns fall into six categories: squat, lunge, hinge, push, pull, and carry [5]. To create an MDL, the fundamental functional training exercises chosen were squats, deadlifts, shoulder presses, lunges, planks, push-ups, pull-ups, and bent-over rows, to understand all the principal movement categories. By using these exercises, it is possible to create more complex exercises like a thruster, which is a a squat followed by a shoulder press, or a burpee, which can consist of different sequences depending on how it is performed: it can consist of a squat followed by a plank and a push-up or begin with a deadlift-like motion that transitions into a plank and push-up. The exact structure can vary between individuals and can be adjusted by the user to match their preferred execution style.

**Figure 1:**
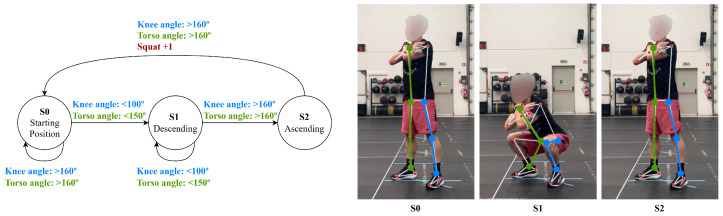
Squat state diagram.

The squat is considered a sequence of three states: the starting position (standing upright), where the athlete stands upright with the knee and torso angles typically above $150^{\circ }$; descending (flexing knees and hips), which is triggered when the knee angle and torso angle decrease below $100^{\circ }$, representing hip and knee flexion into the squat; and ascending (extending to return to the starting position), which is when the knee and torso angles re-extend above $150^{\circ }$ as the athlete returns to the standing position [25], shown in Figure 1.

**Figure 2:**
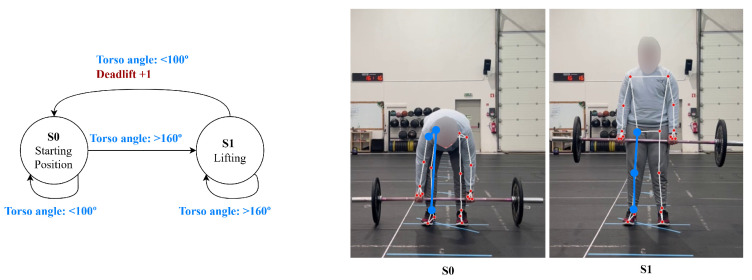
Deadlift state diagram.

The deadlift is divided into two states: state 1, the starting position, where the torso leans forward (with a torso angle below $100^{\circ }$) with straight elbows (above $160^{\circ }$), and state 2, lifting, which is triggered when the torso is extended above $160^{\circ }$ and the elbows remain straight (above $160^{\circ }$), shown in Figure 2.

**Figure 3:**
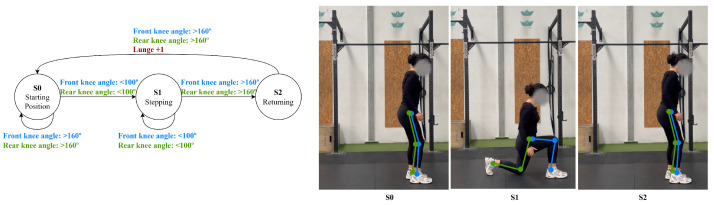
Lunge state diagram.

The lunge has three phases: phase 1, the starting position, where the individual is stood upright, with front and rear knee angles above $160^{\circ }$; phase 2, stepping, where the individual enters the lunge as both knee angles decrease to below $100^{\circ }$; and phase 3, returning, which is the recovery phase as the knee angles are extended again above $160^{\circ }$, shown in Figure 3.

**Figure 4:**
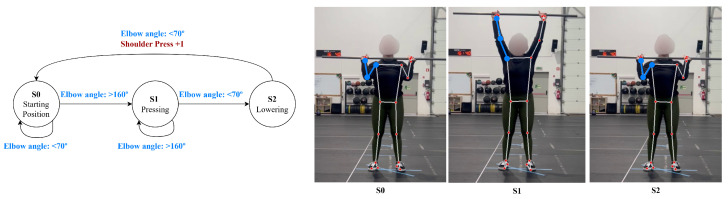
Shoulder press state diagram.

The shoulder press is broken down into three distinct states: the starting position (with the weights at shoulder level), where the elbow and shoulder angles are generally below $70^{\circ }$; the pressing phase (extending the arms upward and lifting the weights overhead), where the elbow and shoulder angles are extended above $160^{\circ }$; and the lowering phase (returning the weights to shoulder level), where the angles return below $70^{\circ }$. During the pressing phase, the system also verifies that the hands and elbows rise above the head, based on the relative position of key points, ensuring correct overhead extension, shown in Figure 4.

**Figure 5:**
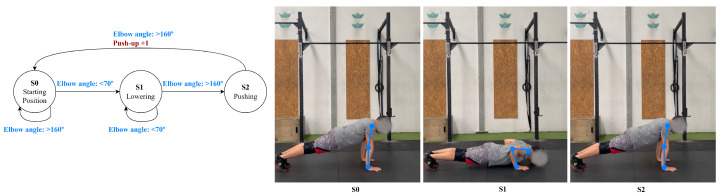
Push-up state diagram.

The push-up is a three-phase cycle: phase 1, the starting position, where the body is in a plank position, and the elbow angle is above $150^{\circ }$; phase 2, lowering, which is triggered when the elbows are bent to below $100^{\circ }$; and phase 3, pushing, where the elbows are re-extended above $150^{\circ }$. In addition, the system verifies that the body remains extended in a horizontal alignment throughout the movement to ensure proper plank posture, shown in Figure 5.

**Figure 6:**
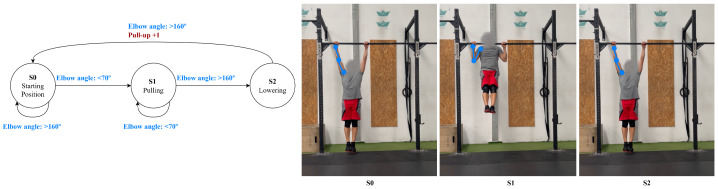
Pull-up state diagram.

For the pull-up, there are three phases: phase 1, the starting position, where the arms are fully extended, the elbow angle is above $160^{\circ }$, and the torso is upright above $160^{\circ }$; phase 2, pulling, where the elbows are flexed below $70^{\circ }$, the chin is moved above the bar, and the torso may lean slightly below $150^{\circ }$; and phase 3, lowering, where the elbows are re-extended above $160^{\circ }$, and the torso returns to an upright position. In addition, the system verifies a vertical displacement of key points, ensuring that the body moves upward and downward along a consistent vertical axis, confirming a proper pull-up motion, shown in Figure 6.

**Figure 7:**
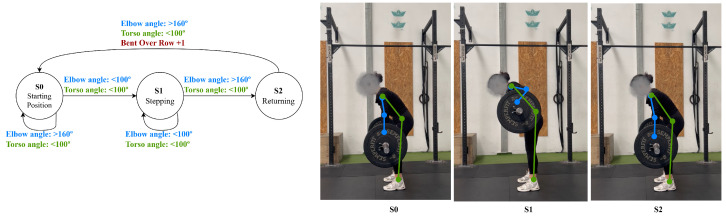
Bent-over row state diagram.

The bent-over row can be divided into three phases: phase 1, the starting position, where the torso is bent forward (with a torso angle below $100^{\circ }$), and the elbows are extended (above $160^{\circ }$); phase 2, pulling, where the elbows are flexed to below 100°, and the weight is lifted; and phase 3, lowering, where the elbows are extended (above 160°) to return to the starting position, shown in Figure 7.

**Figure 8:**
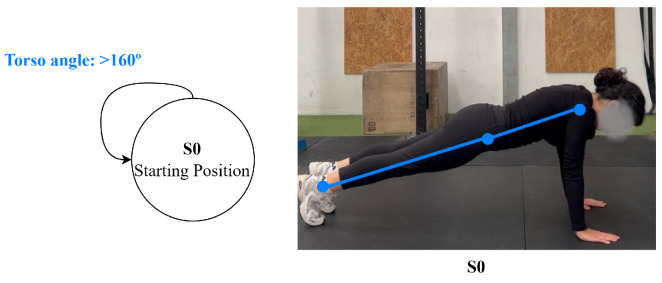
Plank state diagram.

For the plank, which represents isometric exercise modeling, the primary state is a holding position (maintaining body alignment), with specific torso angle thresholds defining proper form maintenance. In addition, the system verifies that the body remains extended in a horizontal alignment throughout the movement to ensure proper plank posture, shown in Figure 8.

**Figure 9:**
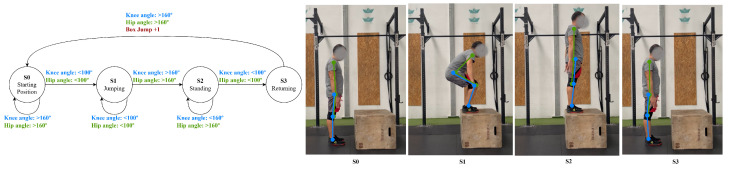
Box jump state diagram.

Lastly, the box jump can be represented in three phases: phase 1, the starting position, where the individual is stood upright, with knee and hip angles above $160^{\circ }$; phase 2, jumping, where the knees and hips are flexed below $100^{\circ }$ before propelling upward; and phase 3, stepping down, where the individual returns to the starting position as the joints are re-extended. To ensure that the jump is correctly recognized and counted, the system also checks for a vertical displacement of the body key points, confirming that the center of mass rises during take-off and lowers during landing, distinguishing a true jump from partial or incomplete movements, shown in Figure 9.

**Figure 10:**
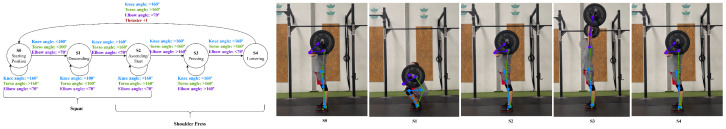
Thruster state diagram.

By defining these primitive components, it is possible to combine them to create more complex exercises. For instance, a thruster can be created by sequencing a squat module immediately followed by a shoulder press module, as shown in Figure 10.

**Figure 11:**
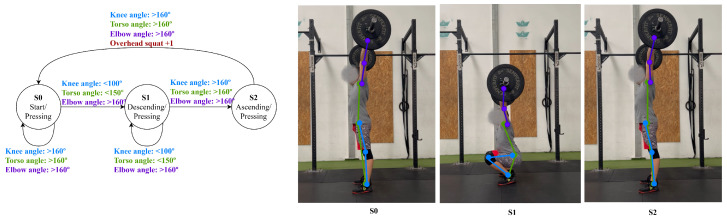
Overhead squat state diagram.

Alternatively, a variant exercise, like an overhead squat, can be created by combining a squat with a shoulder press state, as shown in Figure 11. The specific grip width (for example, a clean grip or snatch grip) affects the shoulder and elbow joint angles, as well as overall stability requirements. The literature shows that wider grip positions (similar to a snatch grip) increase shoulder abduction while decreasing shoulder flexion, whereas narrower grips (like a clean grip) keep the barbell closer to the body, increasing the requirement for shoulder flexion and stability. Moreover, the thoracic posture plays a critical role in shoulder mechanics; a more extended thoracic spine posture enhances scapular posterior tilt and upward rotation, thereby facilitating greater shoulder mobility in overhead positions [26]. The MDL accommodates such variations through user-adjustable angle thresholds, enabling parameterization of exercise definitions for different execution styles without requiring new state machine definitions.

A burpee can be considered a complex exercise that can be described in various ways depending on how it is performed. One common description divides the movement into several phases: starting from a standing position, moving into a plank by placing the hands on the ground and jumping the feet back, performing a push-up, then jumping the feet forward toward the chest, and returning to a standing position with a final jump. Alternatively, a burpee can be performed in a lower-impact variation, where the person steps back into a plank position one foot at a time, performs a push-up, and then steps forward again to stand up without jumping.

**Figure 12:**
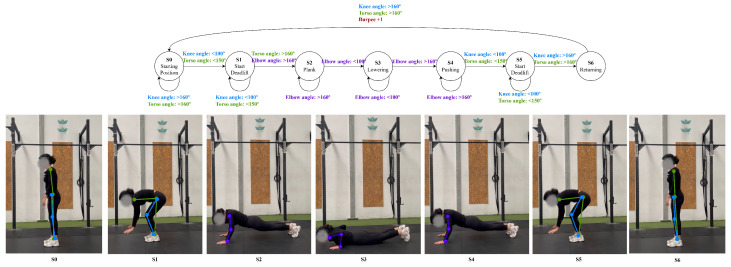
Burpee state diagram.

This movement, as shown in Figure 12, begins from a standing position, followed by lowering the upper body to the starting position of a deadlift as the hips and knees are bent to bring the hands to the ground. From this crouched posture, the legs are extended backwards into a plank, engaging the core and maintaining body alignment. The movement then transitions into a push-up, where the elbows are flexed and extended to lower and raise the torso. After the push-up phase, the athlete jumps or steps their feet forward toward their hands, returning to an upright position through the starting phase of a deadlift, and often finishes with an explosive vertical jump. Throughout the sequence, the burpee integrates the mechanics of the initial pose of a deadlift, followed by a plank, push-up, and jump, making it a complex exercise that develops strength, coordination, and cardiovascular endurance.

The MDL defines a grammar for functional training exercise descriptions, specifying valid syntax for exercise modules, phases, and conditions based on pose-derived angle proxies.
exercise→ “exercise” ID “{” characterization ^+^ “}”characterization→ “characterization” : STRINGphase→ “phase” INTEGER “{” description ${\mathrm{conditions}}^{+}$ “}”description→ “description” : STRINGconditions→ ${\mathrm{condition}}^{+}$condition→ ANGLE_ID COMPARISON NUMBERANGLE_ID→ “knee_angle” | “hip_angle” | “elbow_angle” | “shoulder_angle”COMPARISON→ “>” | “<” | “≥” | “≤”NUMBER→ [0–9]^+^ (“.” [0–9]^+^)?

Each ANGLE_ID represents an angle computed from 3 key points ($k_{i},k_{j},k_{k}$) (where $k_{0}$ = nose, $k_{11}$ = left shoulder, etc.) extracted from MediaPipe’s 33 body landmarks or any pose estimator providing equivalent 3-key-point triplets.

knee_angle $\theta (k_{23},k_{25},k_{27})$—hip-knee-ankle
hip_angle $\theta (k_{23},k_{24},k_{25})$—torso-hip-knee
elbow_angle $\theta (k_{11},k_{13},k_{15})$—shoulder-elbow-wrist
shoulder_angle $\theta (k_{11},k_{12},k_{13})$—neck-shoulder-elbow
torso_angle $\theta (k_{11},k_{23},k_{24})$—neck-hip-torso

where $\theta \left(k_{a},k_{b},k_{c}\right)=\arccos\left(\frac{\vec{k_{b}k_{a}}\cdot \vec{k_{b}k_{c}}}{\left|\right|\vec{k_{b}k_{a}}\left|\right|\cdot \left|\right|\vec{k_{b}k_{c}}\left|\right|}\right)$.

An MDL module represents a single exercise defined as a sequence of movement phases. Each phase is characterized by a set of conditions expressed as constraints on pose-derived angle proxies. A phase is considered active when all its conditions are satisfied. Transitions between phases occur when the corresponding conditions are met, following the natural temporal order of the exercise movement. In this way, each exercise can be modeled as a finite state machine in which phases represent states and the satisfaction of these conditions triggers transitions between them. To illustrate the structure of the MDL, Listing 1 presents an example definition for the squat exercise. The exercise is described through a characterization and a set of phases, where each phase contains constraints on the pose-derived angle proxies used to determine the progression of the movement. The angle values shown in the listing represent default thresholds and are user-adjustable within the graphical user interface (GUI) to accommodate different athletes and execution limitations.

**Listing 1.** Example MDL definition of the squat exercise.

exercise squat {
        characterization:        ‘‘Standing upright, descending by flexing knees and hips,         then returning to the starting position.’’         phase 1 {                  description: ‘‘Starting position’’                  conditions:                knee_angle > 160 # default, adjustable                hip_angle > 160 # default, adjustable        }       phase 2 {                  description: ‘‘Descending’’                 conditions:                knee_angle < 100 # default, adjustable                hip_angle < 150 # default, adjustable      }
}



The MDL definitions are interpreted during video analysis to determine the current phase of the exercise and to count repetitions. Algorithm 1 summarizes the phase detection procedure.
**Algorithm 1:** Phase Detection and Repetition Counting
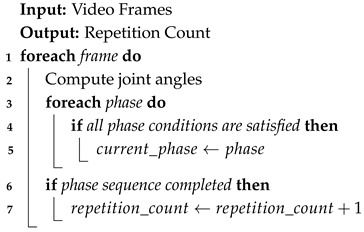


### 2.2. System Architecture and Implementation

**Figure 13:**
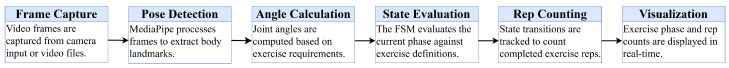
Complete data processing pipeline of the MDL exercise analysis system.

The proposed MDL framework was tested as a real-time, camera-based exercise analysis using pose landmark extraction with finite state machine modeling to enable accurate exercise recognition and repetition counting. The system architecture follows a modular design consisting of three main components: the pose estimation module, the finite state machine engine, and the user interface layer. The angle thresholds can be changed by the user. Figure 13 illustrates the complete camera-based pipeline. For each frame, MediaPipe first estimates 33 body landmarks, which are used to compute pose-derived angle proxies (knee, hip, elbow, shoulder, and torso) used by the MDL definitions. The finite state machine then evaluates these angles against the exercise-specific conditions frame by frame, triggering state transitions and increasing the repetition counter upon completion of a cycle. The GUI provides real-time feedback, displaying phases and repetition counts.

The system uses MediaPipe, a pose estimation framework developed by Google, for camera-based real-time human pose detection and pose-derived angle proxy calculation. It should be noted that MediaPipe landmarks correspond to visually estimated key points. Therefore, the resulting joint angles represent approximate kinematic proxies derived from video. The MDL can process key points extracted by any pose estimation model, with MediaPipe used here for demonstration. MediaPipe provides robust performance across varying lighting conditions, backgrounds, and clothing, making it suitable for diverse training environments. The pose estimation module detects 33 key body landmarks from video frames and calculates pose-derived angle proxies using vector mathematics. Although MediaPipe is used in this implementation, the modular design can accept key points from other pose estimation models.

Pose-derived angle proxies are calculated using the interior angle formula between three points, where three landmarks, A, B, and C, form an angle at point B. The core innovation of the system lies in its finite state machine implementation, which models each exercise as a sequence of movement phases.

The finite state machine operates on real-time pose data, continuously evaluating pose-derived angle proxies against predefined conditions to determine the current exercise phase. State transitions are triggered when angle proxies cross specified thresholds, enabling precise tracking of exercise progression and repetition counting.

A key architectural feature is the configurable threshold system that allows users to customize angle parameters for different exercise variations and execution styles. The default threshold tolerance is set to 30 degrees globally, but users can modify specific angle thresholds for each exercise phase. This flexibility addresses the need for personalized exercise analysis while maintaining the precision required for functional movement assessment.

The threshold configuration is implemented through a hierarchical parameter system: global tolerance settings apply across all exercises, exercise-specific thresholds can override global settings, and individual pose-derived angle proxy limits can be modified for optimal performance.

The system processes video input through a pipeline designed for real-time performance. First, raw video frames are captured from the camera and passed to the pose estimation module, which extracts body landmarks for each frame. These landmarks are then normalized and organized into structured representations that feed the MDL system. The MDL specification encodes each exercise as a finite state machine defined by pose-derived angle proxy thresholds, allowing the system to track exercise phases and count repetitions. The evaluation module applies these rules frame by frame to determine whether the user’s movement satisfies the expected execution criteria and to log relevant events. Finally, the system presents the repetition counts in real time.

In real-time pose estimation, transient frame losses or noisy landmark detections often cause intermittent invalid states, leading the exercise state machine to briefly enter a “None” phase. This behavior can interrupt the logical sequence of movement phases, producing false transitions or missed repetitions. To address this, an adaptive phase persistence mechanism is implemented. Instead of immediately accepting a “None” value when a phase cannot be matched, the system temporarily retains the last valid phase for a short duration of N frames (typically N = 3–5). This persistence window allows the finite state machine to tolerate brief interruptions in pose detection while maintaining continuity in phase tracking. This approach effectively filters out transient tracking errors while preserving responsiveness to genuine phase changes. It is particularly beneficial in movements involving rapid transitions or partial occlusions. And, since the dataset includes videos recorded in wild environments, this adjustment is crucial to keep the state transitions stable despite short tracking interruptions caused by lighting variation, occlusion, or camera movement. Empirical tests showed that adaptive phase persistence eliminated nearly all spurious “None” transitions observed in the initial implementation, reducing false negatives and stabilizing repetition counting.

Adaptive phase persistence retains the last valid phase for up to N frames when no current phase satisfies the transition conditions. If a valid phase is detected before the timeout expires, the state machine resumes normally. Otherwise, the system transitions to “None” and re-evaluates the next frame.

This mechanism acts as a lightweight temporal stability filter, but it is not a full hysteresis model or a low-pass temporal smoother.

The system’s modular architecture enables easy addition of new exercises through standardized exercise definition structures. Each exercise is defined as a separate module containing its specific state machine configuration, making the system extensible and maintainable. The current implementation includes nine fundamental functional training exercises: squats, deadlifts, shoulder presses, lunges, planks, push-ups, pull-ups, bent-over rows, and box jumps.

Complex exercises can be created by combining primitive exercise modules, such as creating a thruster by sequencing a squat module followed by a shoulder press module. This compositional approach enables the representation of advanced functional training routines while maintaining the precision of individual movement analysis.

**Figure 14:**
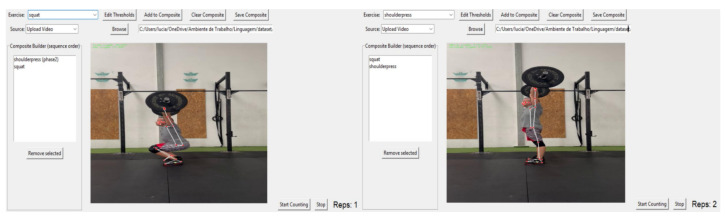
The GUI of the MDL system. **The first image** shows the repetition counting of an overhead squat, corresponding to a squat with the arms extended (shoulder press phase 2). **The second image** illustrates the system counting thruster repetitions, a compound movement consisting of a squat followed by a shoulder press.

The GUI, illustrated in Figure 14, represents the first version of the interface and enables users to intuitively configure exercises, adjust phase thresholds, and visualize results in real time. Through the GUI, users can edit exercise parameters; build composite movements (e.g., squat + shoulder press = thruster or squat + shoulder press (phase 2) = overhead squat), as illustrated; select between webcam or video input, in this case, by uploading a video of an athlete performing thruster repetitions; and monitor real-time repetition counting with live video feedback.

### 2.3. Experimental Evaluation

For experimental validation, a comprehensive dataset was assembled combining publicly available exercise videos and recordings to test the robustness of the Movement Description Language system across diverse exercise scenarios. The dataset was strategically constructed to include multiple exercise types, varying execution qualities, and different environmental conditions to thoroughly evaluate system performance. The evaluation dataset incorporates videos from several established public sources, including exercise demonstration videos from fitness platforms and competition footage. It includes video recordings from extreme conditioning program competitions, which provide high-quality examples of functional training exercises performed under standardized conditions. The links to these videos are available in the shared dataset presented, and they are described later in the article. These competition videos offer valuable ground truth data, as they feature athletes performing exercises with proper form validation by certified judges.

The dataset of extreme conditioning program competition videos includes recordings of fundamental movements such as squats, deadlifts, and other compound exercises that align with the MDL exercise definitions. These videos represent varying skill levels and execution speeds, providing robust test cases for evaluating the system’s ability to recognize exercise phases across different performance qualities and pacing strategies. The dataset includes some of the exercises defined in the MDL framework: squats, deadlifts, shoulder presses, planks, push-ups, and pull-ups. The dataset contains recordings from various settings, including home environments, commercial gyms, and competition venues, testing the system’s robustness to different lighting conditions, backgrounds, and camera angles.

#### 2.3.1. Dataset Collection

The dataset was constructed by collecting videos from public datasets; from extreme conditioning program competitions; and recording videos of 7 participants performing squats, deadlifts, and shoulder presses and 2 participants performing bent-over rows, box jumps, burpees, lunges, push-ups, pull-ups, planks, overhead squats, and thrusters. All participants provided written informed consent to participate in the study and to allow the publication of the research details.

Videos of 9 participants were recorded at the collaborating MAREBox facility following institutional review board approval (CEIC-UA 6-CEIC-UA2026-D). Participants were recruited voluntarily through an open call shared with facility members, and all participants provided written informed consent for participation and publication of the recordings.

The dataset is available at https://1drv.ms/f/c/671361ff08034f89/EjsvyS6EH6lOuhldOcq8t3MByHJ_bomBvAcq06HcV9MiSQ?e=Tz9v2F (accessed on 10 April 2026). All videos were used exclusively for evaluation, as the system does not involve a learning stage. The composition of the dataset across exercises, participants, repetitions, and data sources is summarized in Table 1 and broken down below:

- Recorded Videos (R): 240 videos of squats, 160 videos of deadlifts, 160 videos of shoulder presses, 2 videos of bent-over rows, 4 videos of box jumps, 6 videos of burpees, 3 videos of lunges, 5 videos of push-ups, 4 videos of pull-ups, and 2 videos of plank, thrusters and overhead squats.
- Public Pose Datasets (P): 47 videos of deadlifts, 7 videos of planks, 26 videos of pull-ups, 106 videos of push-ups, 45 videos of shoulder presses, and 7 videos of squats [27,28,29].
- Competition and YouTube Videos (C): 24 videos of deadlifts, 49 videos of shoulder presses, 171 videos of squats, 18 videos of lunges, 6 videos of bent-over rows, 10 videos of box jumps, 7 videos of thrusters, 132 videos of overhead squats, and 3 videos of burpees.

The repetition-counting performance across exercises, including 95% Clopper–Pearson confidence intervals, is summarized in Table 2.

#### 2.3.2. Annotation Procedure

Manual repetition counts served as the ground truth for evaluating the MDL system’s performance on the 689 videos recorded specifically for this study (participant recordings of squats: 240, deadlifts: 160, shoulder presses: 160, bent-over rows: 2, box jumps: 4, burpees: 6, lunges: 3, push-ups: 5, pull-ups: 4, planks: 2, thrusters: 2, and overhead squats: 2; see Table 2).

**Figure 15:**
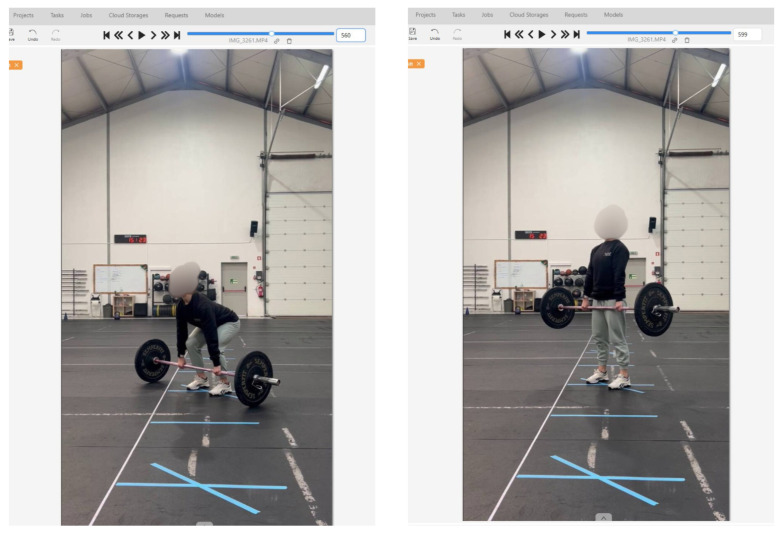
Example of a manual annotation for a deadlift.

Annotations were performed by one annotator using CVAT (https://app.cvat.ai (accessed on 10 April 2026)), an open-source computer vision annotation tool that enables precise frame-level labeling. The labels were created with the names of the exercises. For each video, the start and end frames of individual repetitions were identified based on exercise-specific movement criteria. For the example shown in Figure 15, the start and end of the exercise (in this case, a deadlift) were identified. Each frame within this range was labeled as “deadlift”, from the beginning frame to the end frame, ensuring accurate annotation of the exercise represented. This frame-by-frame approach allowed verification of exercise execution quality and accurate repetition boundary detection.

The annotation task was limited to repetition boundary identification based on clearly defined movement criteria, reducing subjectivity and minimizing ambiguity in labeling.

To evaluate annotation reliability, a subset of three videos from the smartphone recordings dataset was independently annotated by a second annotator. Inter-annotator agreement was assessed at the repetition count level. Repetition count agreement for the same videos revealed identical repetition counts for all sequences (exact match rate of 100%, mean absolute error of 0, Pearson correlation r=1.00, and Cohen’s κ=1.00). These results further demonstrate the consistency of the annotation protocol and the reliability of the manually annotated repetition intervals used for evaluation.

## 3. Results

The repetition counting precision was evaluated by comparing automated repetition counts against manual counts from annotated video sequences. This metric directly assesses the practical utility of the system for fitness applications.

The experimental design includes comparative analysis against existing exercise recognition approaches to contextualize the performance of the MDL-based system. This comparison focuses on accuracy, computational efficiency, and adaptability to new exercise types, highlighting the advantages of the finite state machine approach over traditional machine learning methods [30,31].

The evaluation framework provides comprehensive insights into the practical applicability of the Movement Description Language for real-world functional training analysis, demonstrating its potential for widespread adoption in fitness and rehabilitation applications.

Despite the numerical imbalance between exercise types (e.g., 572 squats versus 12 bent-over rows), the system demonstrated robust repetition counting across all exercises. As shown in Table 2, the MDL framework achieved 100% accuracy in several minority classes, including bent-over rows, burpees, and box jumps. This indicates that the MDL can detect exercise repetitions even with a small set of examples; this is because its rules are based on defined state transitions rather than patterns learned from data, and, therefore, its performance is less sensitive to dataset imbalance than that of learning-based approaches.

Binomial 95% Clopper–Pearson confidence intervals address small sample sizes, as shown in Table 2. Minority classes (bent-over rows n = 12, planks n = 9, and box jumps n = 14) show wider CIs than high-volume exercises (squats and deadlifts), indicating preliminary rule validation rather than definitive generalization.

To assess the effect of adaptive phase persistence, we compared the number of spurious phase resets before and after its introduction. The results show that the mechanism substantially reduced transient “None” transitions and improved repetition stability, particularly in videos with brief occlusions or rapid movement changes. We selected N = 3–5 frames empirically as a short window that removes transient landmark dropouts while preserving responsiveness to genuine phase changes, as shown in Table 3.

The CVAT ground truth provides repetition boundaries rather than phase-level annotations, as the MDL phase definitions are language-specific. We therefore validate phase detection through internal system logging during repetition evaluation: The phase coverage of 98.7% indicates that nearly all counted repetitions included complete MDL phase cycles. The low false transition rate (1.2%) demonstrates robust state machine stability, primarily due to adaptive phase persistence. Since repetitions require sequential phase detection, the 97.2% repetition accuracy validates underlying phase/transition reliability.

Errors were primarily associated with temporary occlusions, rapid transitions, and suboptimal camera viewpoints. These factors led to incorrect or delayed state transitions due to inaccurate pose-derived angle proxies. In most cases, errors resulted in missed repetitions rather than false positives, indicating that the system remains conservative under uncertainty. This was particularly visible in squat and deadlift movements recorded in uncontrolled environments. In these cases, small inaccuracies in pose estimation resulted in pose-derived angle proxy miscalculations that delayed or prevented state transitions in the finite state machine. The quantitative results can be seen in Table 4.

The evaluation dataset contained 1916 ground truth repetitions across 1513 videos, of which 1863 (97.2%) were correctly detected. Accuracy was consistently high across exercise categories, with lunges, pull-ups, and thrusters achieving perfect detection (100%). Slightly lower squat/deadlift performance (95%) resulted from pose estimation noise affecting pose-derived angle proxy calculations.

The computational performance of the proposed system was evaluated using the complete processing pipeline, including pose estimation and MDL-based repetition counting. Experiments were conducted on consumer-grade hardware (Intel/AMD CPU with 16 GB RAM) without GPU acceleration, using videos recorded on a smartphone at 1080p resolution and 30 frames per second. The system achieved 24.6 FPS, corresponding to an average processing latency of 40.6 ms per frame, demonstrating the feasibility of real-time exercise analysis. The computational overhead introduced by the MDL finite state machine was negligible, requiring only a few microseconds per frame for state evaluation and transition logic. Consequently, the overall computational cost was dominated by the pose estimation stage, while the MDL-based repetition detection contributed minimally to the total processing time.

To compare the MDL against established repetition counting methods, RepNet, the current state-of-the-art class-agnostic approach [15], was selected as a baseline. RepNet was chosen as the baseline because it is a widely used, publicly available, class-agnostic approach specifically designed for repetition counting in video. Its temporal self-similarity formulation makes it a strong and reproducible reference for evaluating the MDL on the same dataset. To the best of our knowledge, no publicly available implementations of rule-based FSM approaches for functional training exist with standardized evaluation protocols, limiting direct comparisons beyond RepNet. Table 5 presents the repetition counting accuracy of RepNet on the evaluation dataset.

## 4. Discussion

The results demonstrate that the proposed MDL provides a structured and extensible framework for representing and analyzing functional training exercises. The accuracy achieved across all fundamental and compound exercises—averaging above 95%—confirms that finite state modeling combined with real-time pose estimation can effectively capture exercise dynamics and transitions.

The MDL can be used as an alternative to machine learning approaches that require large labeled datasets for each exercise; it enables transparent, interpretable, and reusable definitions of exercise logic. Each exercise can be expressed as a modular state machine, facilitating debugging, adaptation, and composition into more complex routines such as thrusters and overhead squats. The success of these compound movements highlights the expressiveness of the MDL’s compositional syntax: by combining previously defined primitives (e.g., squat + shoulder press), new exercises can be recognized without extending the MDL, simply by using the exercises already defined or by combining phases of specific exercises to detect and count repetitions of new exercises.

It is important to note the significant class imbalance in the evaluation dataset, where certain fundamental exercises like squats and deadlifts are represented by hundreds of videos, while others are represented by fewer than ten. In traditional data-driven or black-box recognition systems, such an imbalance often leads to bias toward majority classes, compromising the reliability of the reported accuracy. However, because the MDL is a rule-based system grounded in explicit biomechanical logic and pose-derived angle proxy transitions, its performance is inherently independent of the number of training samples. The fact that the system maintained an average accuracy above 95% across both high-volume and low-volume exercises confirms its robustness and low data dependency.

While several low-sample exercises achieved 100% accuracy, their Clopper–Pearson 95% CIs remained wider (planks [69.2–100%] and bent-over rows [78.2–100%]) than those of high-volume exercises (squats [92.7–97.4%]). Although the MDL’s performance is training-independent, evaluation certainty remains limited by sample size. These results validate rule correctness for observed cases rather than establishing population-level generalization bounds. A key improvement implemented during this work was the stabilization of state detection. Earlier issues, such as transient “None” states caused by temporary occlusions or tracking noise, were successfully mitigated by introducing adaptive phase persistence. This allowed the system to maintain continuity through short interruptions in pose estimation, resulting in smoother transitions and more reliable repetition counting.

However, few errors were detected, and those that were detected mainly resulted from inaccurate key point tracking. MediaPipe’s performance can degrade when the subject is partially occluded or moves too quickly or when camera angles deviate from frontal or lateral views. In such cases, small landmark inaccuracies propagate into pose-derived angle proxy miscalculations, leading to incorrect or delayed state transitions. While adaptive phase persistence masks short interruptions, it cannot correct fundamentally misplaced landmarks.

A fundamental limitation of monocular pose estimation systems is that detected landmarks do not correspond to anatomical joint centers and are affected by factors such as occlusion, perspective distortion, and soft tissue motion. Consequently, the pose-derived angle proxies derived from these landmarks should be interpreted as approximate kinematic indicators rather than precise biomechanical measurements. The MDL framework processes key points from any pose estimation model and supports more accurate motion capture systems.

**Figure 16:**
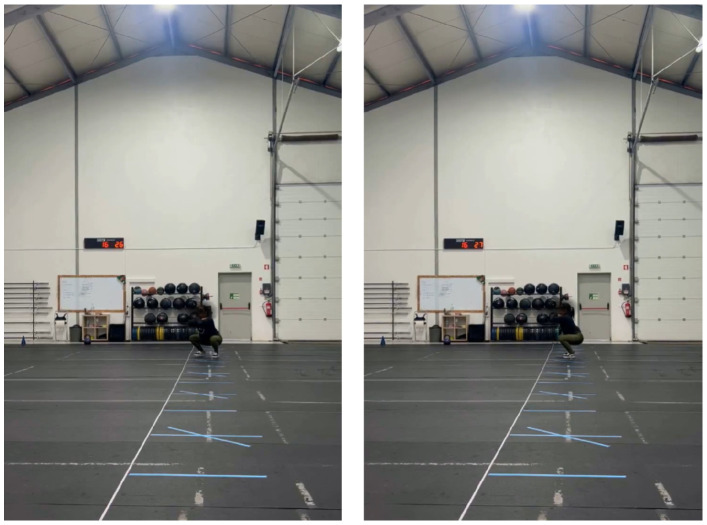
Example of dark clothing blending with the background.

The occlusion of key body parts was noticed particularly during exercises that involve bending movements such as squats and deadlifts. These occlusions were more pronounced at greater distances, making it difficult for the system to maintain accurate landmark detection. In addition, joint misalignment and errors in pose estimation were observed when participants wore dark clothing that blended with the background; Figure 16 shows an example.

This affected the state transitions in the finite state machine because not all the phases were correctly identified, and, as a result, the correct detection of a repetition was compromised.

Compared to deep learning-based exercise recognition systems, the MDL framework provides a more transparent and customizable alternative. Table 6 compares the MDL against machine learning approaches, demonstrating equivalent repetition counting accuracy using explicit phase logic rather than data-driven training. Each decision follows deterministic finite state logic, enabling full interpretability and user customization of movement definitions. Unlike data-driven models, which require large annotated datasets and often act as opaque “black boxes”, the MDL operates effectively without extensive training data. Furthermore, because the system allows users to adjust pose-derived angle proxy thresholds according to their individual mobility limitations while maintaining values within valid ranges, it can adapt to different users in a way that remains explainable and physiologically meaningful. Although machine learning approaches may generalize better across uncontrolled scenarios, the MDL’s configurability, clarity, and low data dependency make it a robust and practical solution for real-world functional training analysis. It should be noted that the comparison with machine learning approaches is conceptual and based on reported results in the literature, as no shared benchmark dataset exists for direct evaluation. However, reported accuracies for skeleton-based action recognition methods in exercise scenarios typically range between 85% and 95%, often requiring large labeled datasets and model retraining for new exercises. In contrast, the MDL achieves comparable repetition counting accuracy without any training phase, relying instead on explicit and interpretable movement definitions.

The MDL relies on kinematic data (pose-derived angle proxies). Although sufficient for accurate repetition detection and phase identification, pose-derived angle proxies alone cannot capture muscular effort, fatigue, or inter-user biomechanical differences. To advance toward personalized and physiologically grounded movement analysis, future work should focus on expanding the validation dataset to include a more balanced and diverse range of exercises and participants. While the current rule-based approach proves effective with small sample sizes, testing on a larger scale will be essential to further refine pose-derived angle proxy thresholds and ensure the system’s generalizability across even more varied execution styles and uncontrolled environments, as well as integrating additional data modalities such as electromyography (EMG), velocity, and force estimates. These would enable the language to represent not only motion but also the intention and intensity behind it.

The evaluation was conducted on a dataset composed mainly of healthy adults performing functional training exercises in gym, competition, and home environments. Although this diversity of recording conditions supports validity for sports and fitness applications, it also constrains the generalizability of the findings. Movement strategies, joint ranges, and compensatory patterns in individuals with limitations or patients undergoing rehabilitation may differ substantially from those observed in this dataset, and they may not always conform to the pose-derived angle proxy thresholds and phase definitions used in the current MDL configuration. However, these populations also introduce clinical risks: automated systems may incorrectly classify compensatory movements as valid repetitions (encouraging poor technique) or reject legitimate patient-adapted movements due to overly strict thresholds. At the same time, a key advantage of the MDL framework is that these thresholds are configurable: clinicians or practitioners can lower or narrow the acceptable angle ranges to reflect a reduced range of motion or specific movement limitations while preserving the underlying phase structure of the exercise. Thus, although the present validation was conducted in healthy individuals, the language was designed so that its parameters can be adapted to individuals with restrictions, which is an important focus for future rehabilitation-oriented studies.

The MDL framework demonstrates robust repetition counting (>95% accuracy across exercises), tested with MediaPipe landmark extraction. MediaPipe is known to be sensitive to camera viewpoint, occlusion, and lighting conditions, though it performs reliably under typical gym conditions [34], delivering smooth real-time feedback consistent with its design for live operation. The few remaining errors (5% overall) primarily occur during partial occlusions, rapid limb movements (>5 m/s), or when camera angles deviate substantially from frontal/lateral views [34], reflecting known pose estimation limitations rather than MDL logic failures. Although no direct comparison with motion capture systems was performed, the high repetition counting accuracy and the high phase coverage suggest that the pose-derived signals are sufficiently stable to support consistent state transitions. Since repetition detection requires coherent phase sequencing, these results indirectly validate the robustness of the underlying pose-derived representations for the intended application.

Overall, the MDL demonstrates a balance between interpretability, flexibility, and precision. It serves as a bridge between low-level sensor data and high-level exercise semantics, creating opportunities for more adaptive and intelligent movement analysis systems.

## 5. Conclusions

This study demonstrates that functional training exercises can be modeled through an executable and composable representation that bridges human-readable definitions and real-time motion analysis. The proposed MDL differs from existing approaches by combining three properties that are not jointly supported in prior work: (i) executable semantics enabling direct deployment without post-processing or interpretation layers, (ii) a compositional structure allowing new exercises to be defined through modular combinations of primitives, and (iii) independence from data-driven training, enabling immediate generalization to unseen exercise compositions. These characteristics position the MDL as a complementary alternative to data-driven recognition systems, particularly in scenarios where interpretability, configurability, and low-data deployment are required.

Testing with MediaPipe’s real-time pose estimation confirmed the language’s practical utility. The results show that both simple and compound exercises, including thrusters and overhead squats, can be automatically recognized using only existing building blocks, without requiring any modification or extension of the MDL. This demonstrates the compositional power of the language: new exercises can be represented through logical combinations of existing primitives rather than by defining new syntax or structures.

The addition of adaptive phase persistence proved essential for handling temporary pose estimation losses, significantly improving stability in real-world conditions. This refinement demonstrates the maturity of the current system and sets the foundation for multimodal extensions. Empirical evaluation confirms that this mechanism substantially reduced false “None” transitions and improved repetition accuracy across all exercises.

Future work will incorporate additional biomechanical and physiological signals, including those from wearable sensors for heart rate, EMG data, and movement velocity derived from video analysis, to complement the existing kinematic data. Enhanced velocity-based training metrics will enable precise, context-aware feedback aligned with established sports science practices. Automatic landmark quality assessment will further ensure robust performance across diverse users and exercises. At the same time, future work should also focus on quantitatively evaluating the accuracy of MediaPipe’s pose-derived angle proxies against that of gold-standard motion capture, systematically analyze performance under controlled camera/lighting variations, and measure explicit end-to-end latency from camera capture to repetition count output. These pose estimation analyses will address current limitations, such as the inability to automatically correct misidentified landmarks and known sensitivity to viewpoint/occlusion to ensure that assessments remain accurate and reliable across users and exercises. And while the current implementation relies on monocular pose estimation, future work will focus on validating the framework using higher-precision biomechanical measurement systems. Together, these enhancements will enable richer and more detailed movement characterization; facilitate adaptive, user-specific thresholding through learning algorithms; and open new possibilities in areas such as performance optimization, injury prevention, and personalized exercise guidance.

The MDL framework provides opportunities for automated movement documentation and generation in addition to real-time analysis. Exercises can be dynamically generated, altered, and interpreted by large language models (LLMs) due to their structured, machine-readable definitions. This enables systems that can automatically record workouts, produce new exercise models from textual prompts, or update preexisting definitions based on user feedback by bridging the gap between executable MDL code and natural language descriptions of exercises. Through human–AI cooperation, the MDL could develop into a living repository of human movement knowledge that is constantly added to, improved upon, and validated.

In summary, the MDL provides a structured and interpretable framework for representing and detecting functional exercise phases using video-based pose estimation, combining the precision of computational modeling with the adaptability needed for real-world functional training.

## Figures and Tables

**Table 1:** Summary of the dataset composition by exercise type, including number of videos, annotated repetitions, participants involved in the recordings performed at the MAREBox facility, and data sources. R: recorded at MAREBox facility; P: public pose datasets; C: competition and online videos.

Exercise	Videos	Reps	Participants	Source	Distribution (%)
Squat	418	572	7 + 31 + 10 = 48	R + P + C	29.8
Deadlift	231	379	7 + 16 + 10 = 33	R + P + C	19.8
Lunge	21	42	2 + 10 = 12	R + C	2.2
Shoulder Press	254	356	7 + 16 + 10 = 33	R + P + C	18.6
Push-Up	111	238	2 + 14 = 16	R + P	12.4
Pull-Up	30	94	2 + 22 = 24	R + P	4.9
Bent-Over Row	8	12	2 + 2 = 4	R + C	0.6
Plank	9	9	2 + 7 = 9	R + P	0.5
Box Jump	14	14	2 + 2 = 4	R + C	0.7
Thruster	9	33	2 + 7 = 9	R + C	1.7
Overhead Squat	134	141	2 + 22 = 24	R + C	7.4
Burpee	9	26	2 + 2 = 4	R + C	1.4
Total	1248	1916	9 + 106 + 43 = 158	R + P + C	100

**Table 2:** Accuracy of repetition counting in the evaluation dataset with 95% Clopper–Pearson confidence intervals across exercises.

Exercise	Reps	MDL Reps	Accuracy (%)	95% CI
Squat	572	546	95%	[92.7–97.4%]
Deadlift	379	360	95%	[92.4–96.8%]
Lunge	42	42	100%	[91.8–100%]
Shoulder Press	356	355	100%	[98.3–100%]
Push-Up	238	234	98%	[95.9–99.6%]
Pull-Up	94	94	100%	[96.4–100%]
Bent-Over Row	12	12	100%	[78.2–100%]
Plank	9	9	100%	[69.2–100%]
Box Jump	14	14	100%	[81.6–100%]
Thruster	33	33	100%	[89.4–100%]
Overhead Squat	141	138	98%	[93.8–99.6%]
Burpee	26	26	100%	[89.0–100%]
Total	1916	1863	97.2%	[96.0–98.2%]

**Table 3:** Effect of adaptive phase persistence on phase stability and repetition counting.

Setting	False Transitions	Repetition Accuracy
Without persistence	337 errors	82.4%
With persistence (N = 3–5)	53 errors	97.2%

**Table 4:** Quantitative error distribution by exercise type.

Exercise	Errors	Error Rate	Main Causes
Squat	26	4.5%	Bottom position miss (26)
Deadlift	19	5.0%	Angle threshold (7), bottom position miss (12)
Push-Up	4	1.7%	Chest proximity failure (3), arm occlusion (1)
Overhead Squat	3	2.1%	Bottom position miss (3)
Others (8 exercises)	0	0%	-
Total	53	2.8%	

**Table 5:** Repetition counting performance: RepNet baseline vs. proposed MDL framework. GT = ground truth repetitions.

Exercise	GT	Reps RepNet	Acc RepNet (%)	Reps MDL	Acc MDL (%)
Squat	572	433	76	546	95
Deadlift	379	278	73	360	95
Lunge	42	16	38	42	100
Shoulder Press	356	294	83	355	100
Push-Up	238	178	75	234	98
Pull-Up	94	17	18	94	100
Bent-Over Row	12	4	33	12	100
Plank	9	1	11	9	100
Box Jump	14	4	29	14	100
Thruster	33	19	58	33	100
Overhead Squat	141	86	61	138	98
Burpee	26	8	31	26	100
Total	1916	1364	71.2	1863	97.2

**Table 6:** Comparison of exercise recognition approaches.

Approach	Example Methods	Avg. Accuracy	Dataset Type/Size	Key Traits	Limitations
MDL	Pose-derived angle proxy thresholds, MediaPipe	95–100%	Custom functional videos (∼1300, real world)	Interpretable; no training data; composable; real time	Pose estimation quality-dependent; manual tuning for variants
DL (CNN/LSTM)	BiLSTM w/joint angles [30], CNN posture [32]	91–99% (strength 91%, HIIT 86%)	Mixed videos (Kaggle, InfiniteRep) [30,32]	Temporal dynamics; transfer learning	Large data needs; black box; env. variability
ML (Skeleton)	LSTM skeletons [33], BiLSTM class [30]	90–95% rep counting	Kaggle workouts, real-time videos [30,33]	Real time; angle invariance	Frame issues; data bias; poor cross-dataset performance

## Data Availability

The datasets generated and analyzed for this study can be found here https://1drv.ms/f/c/671361ff08034f89/EjsvyS6EH6lOuhldOcq8t3MByHJ_bomBvAcq06HcV9MiSQ?e=Tz9v2F (accessed on 10 April 2026).

## Abbreviations

The following abbreviations are used in this manuscript:

MDL	Movement Description Language
FT	Functional Training
EWMN	Eshkol-Wachman Movement Notation
FMS	Functional Movement Screen
UML	Unified Modeling Language
GUI	Graphical User Interface
LLM	Large Language Model
